# NOX1 and NOX4 are required for the differentiation of mouse F9 cells into extraembryonic endoderm

**DOI:** 10.1371/journal.pone.0170812

**Published:** 2017-02-02

**Authors:** Benjamin J. Dickson, Mohamed I. Gatie, Danielle M. Spice, Gregory M. Kelly

**Affiliations:** 1 Department of Biology, Molecular Genetics Unit, Western University, London, Ontario, Canada; 2 Child Health Research Institute, London, Ontario, Canada; 3 Ontario Institute for Regenerative Medicine, Toronto, Ontario, Canada; Duke University, UNITED STATES

## Abstract

Mouse F9 cells differentiate to primitive endoderm (PrE) when treated with retinoic acid (RA). Differentiation is accompanied by increased reactive oxygen species (ROS) levels, and while treating F9 cells with antioxidants attenuates differentiation, H_2_O_2_ treatment alone is sufficient to induce PrE. We identified the NADPH oxidase (NOX) complexes as candidates for the source of this endogenous ROS, and within this gene family, and over the course of differentiation, *Nox1* and *Nox 4* show the greatest upregulation induced by RA. *Gata6*, encoding a master regulator of extraembryonic endoderm is also up-regulated by RA and we provide evidence that NOX1 and NOX4 protein levels increase in F9 cells overexpressing *Gata6*. Pan-NOX and NOX1-specific inhibitors significantly reduced the ability of RA to induce PrE, and this was recapitulated using a genetic approach to knockdown *Nox1* and/or *Nox4* transcripts. Interestingly, overexpressing either gene in untreated F9 cells did not induce differentiation, even though each elevated ROS levels. Thus, the data suggests that ROS produced during PrE differentiation is dependent in part on increased NOX1 and NOX4 levels, which is under the control of GATA6. Furthermore, these results suggest that the combined activity of multiple NOX proteins is necessary for the differentiation of F9 cells to primitive endoderm.

## Introduction

Mouse extraembryonic endoderm formation can be studied *in vitro* using F9 teratocarcinoma cells, which are chemically induced to form primitive endoderm (PrE) when treated with retinoic acid (RA) [1] or to parietal endoderm (PE) when treated with RA and cAMP analog [2]. F9 cell differentiation, specifically to PrE is accompanied by the appearance of molecular markers, and morphological changes, many resulting from the activation of the canonical Wnt-β-catenin pathway [1]. In this pathway when Wnt is absent a destruction complex serves to phosphorylate β-catenin marking it for ubiquitination and degradation in the proteasome. When present, Wnt binds to a Frizzled receptor causing Dishevelled (DVL) to move towards the plasma membrane, where it recruits Axin out of the destruction complex making it non-functional and allowing β-catenin to accumulate and translocate to the nucleus where it binds to and activates the T-cell-factors-Lymphoid enhancer factors (TCF-LEF) family of transcription factors. We reported previously that differentiation is also accompanied by a burst of ROS, which is necessary as F9 cells treated with antioxidants or when treated with a non-specific NADPH oxidase inhibitor failed to form PrE [3]. That H_2_O_2_ treatment alone induces PrE indicates that ROS are sufficient to initiate differentiation [3]. To explore this further, we recently reported that DVL in undifferentiated F9 cells associates with nucleoredoxin (NRX) a redox sensitive protein that scavenges ROS, and is known to play a role in PrE differentiation [4]. This association and regulation of the Wnt-β-catenin pathway occurs in other systems [5–8], and we propose that this inhibition prevents aberrant canonical Wnt signaling when Wnt is absent as DVL in this state cannot recruit Axin away from a destruction complex. Thus, in the presence of ROS NRX dissociates from DVL and the Wnt pathway is primed awaiting the ligand.

The source of the ROS when F9 cells are treated with RA was investigated and the candidates identified are members of the NADPH oxidase (NOX) family, which are sources of superoxide anions and H_2_O_2_ [9]. In F9 cells *Nox1-4 and Duox2* are upregulated following RA treatment [3]. *Duox1* is not RA-responsive and may not be involved in PrE differentiation. *Nox1* and *Nox4* are up-regulated to the greatest extent following RA treatment, and given the previous reports suggesting a link to extraembryonic endoderm formation and stem cell differentiation, we specifically selected these members to interrogate as the candidates involved in the ROS production involved in RA-induced PrE formation. To address that the activity of NADPH oxidase 1 and/or 4 is/are responsible for producing the ROS that are necessary and sufficient to induce F9 cells to differentiate, we first tested and found Nox genes are under the control of GATA6, the master regulator of endoderm differentiation [10]. Inhibiting all NOX activity, or specifically inhibiting NOX1 was sufficient to block differentiation, and knocking down *Nox1* or *Nox4* expression using an siRNA approach complemented the chemical inhibitor data. Confident from these studies that both NOX proteins were necessary for differentiation, we expected that their overexpression would induce PrE. However, despite the overexpression of each having increased ROS levels, no significant difference in β-catenin-dependent TCF activity relative to controls was seen and neither would induce PrE. Together, these results indicate that RA-induced differentiation of F9 cells requires a coordinate increase in NOX activity that is due in part to the upregulation of the *Nox* genes by GATA6.

## Materials and methods

### Cell culture conditions and transfections

Mouse teratocarcinoma F9 cells (ATCC) were cultured in Dulbecco’s modified Eagles medium (Lonza) supplemented with 10% fetal bovine serum (Gibco) and 1% penicillin-streptomycin (Lonza), and incubated at 37°C and 5% CO_2_. Cells were treated with 10^−7^ M retinoic acid (RA all-trans; Sigma Aldrich) or dimethyl sulfoxide (DMSO; Caledon) as a negative control. Cells were co-treated with 1 μM VAS2870 (Sigma) and RA 24 hours after seeding and grown for 3 days, or co-treated with 250 nM ML171 (Tocris) and RA and grown for 4 days as described above. F9 cells were reverse transfected using Lipofectamine 2000 (Thermo Fisher Scientific). Freshly passaged cells were added to a 35 mm dish already containing a total of 4 μg of DNA plasmid. Culture media was replenished 6–8 h post-transfection and transfected cells were selected using antibiotics.

### Plasmids

The following plasmids were used: pcDNA3.1-*Gata6;* pcDNA*-mNox4*, a gift from Dr. M. Jaconi, University of Geneva), pcDNA3.1-*mNox1* (Addgene # 58340), pRL-*TK*, a gift from Dr. R. DeKoter (Western University), piLenti-siRNA-GFP-*Nox1* “CTATTTAACTTCGAACGCTACAGAAGAAG”, piLenti-siRNA-GFP-*Nox1* “TGCTTCCATCTTGAAATCTATCTGGTACA”, piLenti-siRNA-GFP-*Nox4* “ACATTTGGTGTCCACTTTAAAGTAGTAGG”, piLenti-siRNA-GFP-*Nox4* “TCCAGTGGTTTGCAGATTTACTCTGTGTG” (Applied Biological Materials, Richmond BC).

### Immunoblot analysis

Protein lysates were collected in RIPA buffer containing 150 mM sodium chloride, 1.0% Triton X-100, 0.5% deoxycholate, 0.1% SDS and 50 mM Tris pH 8.0. Protein concentrations were determined using the DC^™^ Protein Assay (Bio-Rad), and 20–50 μg of protein lysate was mixed 2:1 with 3X SDS loading buffer containing 10% β-mercaptoethanol and separated on 10% polyacrylamide gels by electrophoresis for 2 h with 100 V at 4°C. Following electrophoresis, the proteins were wet-transferred electrophoretically to Immunoblot PVDF membrane (Bio-Rad) for 16 h with 20 V at 4°C using a Tris-glycine transfer buffer containing 20% methanol. Membranes were incubated in Tris buffered saline with 0.1% Tween-20 (TBS-T) containing 10% w/v skim milk powder for 1 h shaking at room temperature, then incubated with primary antibody overnight at 4°C. Following 3 washes 5 min each in TBS-T, membranes were incubated with secondary antibody for 2 h at room temperature, followed by 3 washes for 10 min each in TBS-T. SuperSignal West Pico Chemiluminescent Detection Kit (Thermo Fisher Scientific) was used to detect the presence of secondary antibodies conjugated to horseradish peroxidase (HRP). Signals were captured using a Molecular Imager Gel Doc XR system (Bio-Rad) with Quantity One Software. The primary antibodies used were directed against TROMA1 (1:10; 55 kDa, Developmental Studies Hybridoma Bank), OCT4 (1:1000; 42 kDa, Cell Signaling Technology), DAB2 (1:10000; 96 kDa, BD Transduction Laboratories), NOX1 (1:10000; 65 kDa, Thermo Fisher Scientific), NOX4 (1:10000; 67 kDa, Novus Biologicals) and ß-actin (1:10000; 47 kDa, Santa Cruz) dissolved in 3% Bovine Serum Albumin w/v in TBS-T. Secondary anti-rat (1:1000), anti-mouse (1:1000) and anti-rabbit (1:1000) antibodies were HRP-conjugated and dissolved in 3% skim milk w/v in TBS-T.

### Quantitative reverse transcription polymerase chain reaction

Total RNA from treated and/or transfected cells was isolated and collected using the RNeasy Mini Kit (Qiagen). RNA was reverse transcribed into first strand cDNA using the High-Capacity cDNA Reverse Transcription Kit (Applied Biosystems) and the manufacturer's recommendations. The CFX Connect Real-Time PCR Detection System (Bio-Rad) was used for qRT-PCR analysis. Each reaction contained 500 nM of each primer, SensiFAST SYBR Mix (FroggaBio), and 1 μL of cDNA. Primers were designed to: *L14* F (5’GGGAGAGGTGGCCTCGGACGC), *L14* R (5’GGCTGGCTTTCACTCAAAGGCC), *Gata6* F (5’ATGGCGTAGAAATGCTGAGG), *Gata6* R (5’TGAGGTGGTCGCTTGTGTAG), *Dab2* F (5’GGAGCATGTAGACCATGATG), *Dab2* R (5’AAAGGATTTCCGAAAGGGCT), *Nox1* F (5’AATGCCCAGGATCGAGGT), *Nox1* R (5’GATGGAAGCAAAGGGAGTGA), *Nox4* F (5’GATCACAGAAGGTCCCTAGCA), and *Nox4* R (5’GTTGAGGGCATTCACCAAGT). Analysis of gene expression was determined using the comparative cycle threshold (Δ/ΔCt) method. Gene expression was normalized to the constitutively expressed *L14* gene and relative values were normalized by comparing treatments to DMSO-treated and/or control plasmid transfected control cells to determine fold change.

### TCF reporter assay

Cells were transfected with pGL3*-BARL* and pRL*-TK* (transfection control) and then treated with DMSO (vehicle control), RA, or co-transfected with *Nox1* or *Nox4* plasmids in equal amounts of DNA. Protein lysates were collected 3 days post-treatment or post-transfection using the Dual-Glo Luciferase Assay System and the manufacturer's recommendations (Promega). Luciferase expression was quantified using the GloMax Multi Detection System (Promega). Values were presented as relative luminescence derived from the quotient of firefly luminescence (pGL3*-BARL*) and Renilla luminescence (pRL*-TK*).

### ROS detection

Intracellular ROS was detected using both an Amplex Red Assay and CM-H_2_DCFDA (Thermo Fisher Scientific). Briefly, Amplex Red was prepared in DMSO and cells were treated following manufacturer’s instructions with slight modifications. Following treatment or transfection, cells were detached and resuspended in 100 μM Amplex Red in measurement media buffer (120 mM KCl, 5 mM KH_2_PO_4_, 5 mM EDTA, 10 mM HEPES, 5 mM MgCl_2_ at pH 7.40) supplemented with 40 μg/mL Saponin (Sigma) and 0.2 units/mL HRP (Thermo Fisher Scientific). Immediately after incubation, cells were seeded onto an opaque 96-well plate and florescence was measured at Ex/Em of 530/590 nm using the GloMax Multi Detection System (Promega). Values were normalized to protein concentration. For CM-H_2_DCFDA staining cells following treatment with DMSO or RA were incubated in Hank’s Balanced Salt Solution for 15 min at 37°C and 5% CO_2_ with 2 μM CM-H_2_DCFDA dissolved in DMSO and 10 μg/ml Hoechst 33342 (Thermo Fisher Scientific). Immediately after incubation, cells were rinsed twice with PBS and images were captured using a Zeiss Axio Observer A1 inverted microscope with a QImaging Retiga CCD.

### MTT assay

F9 cells were cultured and treated with DMSO, RA, VAS2870 or ML171 for 4 days followed by incubation in MTT reagent (Sigma) for 2–4 hours at 37°C and 5% CO_2_. Media was removed and DMSO was added to solubilize crystals, and then plates were incubated overnight in the dark. Absorbance values were measured at 570nm with a reference wavelength at 650nm using the GloMax^®^-Multi Detection System (Promega).

### Statistical analysis

Data from qRT-PCR, densitometric analysis of immunoblots and Luciferase assays were gathered format least three independent biological replicates. Comparisons of data between control and experimental groups were performed using a one-way ANOVA with Tukey’s honest significant difference (HSD) post-hoc test or a Student’s t-Test (SPSS Statistics for Windows Version 19.0, IBM Corp. Released 2010, Armonk, NY). Student’s t-Test was used for statistical analysis of data when comparing control to only one experimental data set and (*) was used to denote significant difference. All other data were analyzed using a one-way ANOVA followed by Tukey’s HSD test and letters were used to indicate significant differences. P-values were considered statistically significant at the 0.05 level. Statistical data are presented as the mean ± SEM.

## Results

### RA increases NOX1 and NOX4 levels and ROS production

**Fig 1:**
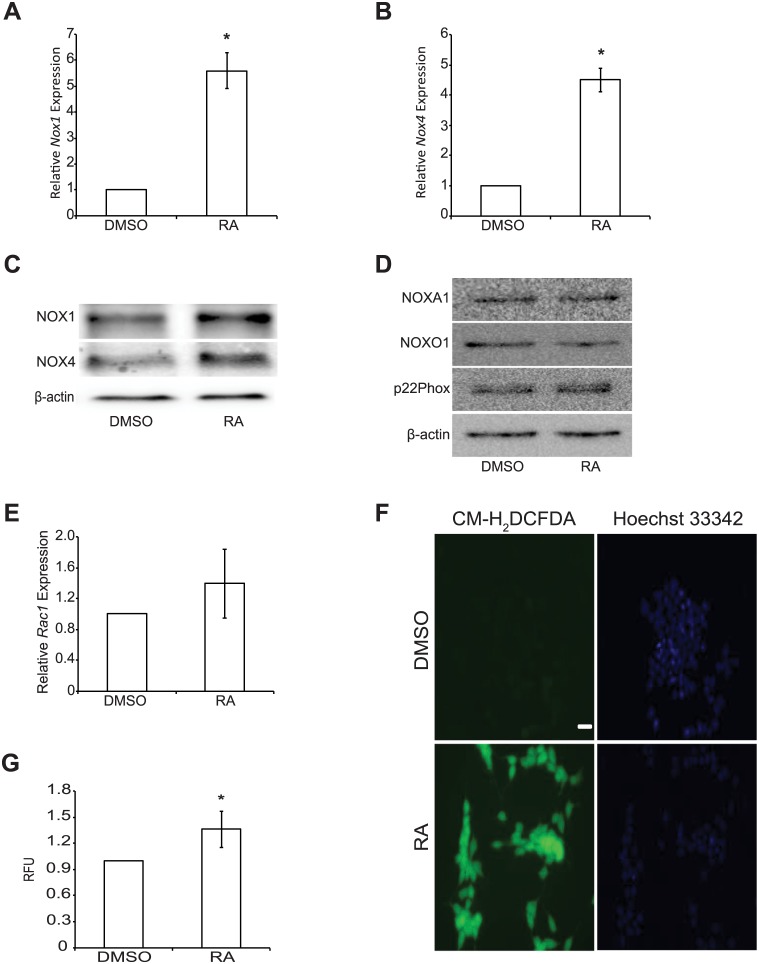
RA increases NOX1 and NOX4 levels and ROS production. Total RNA and protein was harvested from F9 cells treated with either DMSO or RA for 4 days. **(A)** *Nox1* and **(B)** *Nox4* expression following DMSO or RA treatment. (**C**) NOX1 and NOX4 protein levels following DMSO and RA treatment. **(D)** Protein levels of NADPH oxidase complex accessory subunits NOXA1, NOXO1, and p22Phox following DMSO and RA treatment. **(E)** *Rac1* expression following DMSO or RA treatment. **(F)** ROS detection using CM-H_2_DCFDA or **(G)** Amplex Red in F9 cells treated with either DMSO or RA. A total of 3–5 independent experiments were analyzed and results presented as mean ± SEM. * denotes significance (*P* < 0.05) tested by a Student’s t-Test.

F9 cells treated with RA for 4 days showed a significant increase (*P* < 0.05) in the expression of *Nox1* (Fig 1A) and *Nox4* (Fig 1B), as we have shown previously [3]. Furthermore, NOX1 and NOX4 protein levels also increased after RA treatment (Fig 1C), however, the levels of accessory subunits required for NOX protein function, including NOXA1, NOXO1 and p22Phox (Fig 1D), or the transcripts encoding *Rac1* (Fig 1E), did not appear to change during differentiation. Thus, the subunits to generate functional NOX1 and NOX4 were present in undifferentiated F9 cells. The transcripts encoding the NOX subunits, however, are in low abundance prior to differentiation to PrE (S1A–S1C Fig). More importantly, elevated ROS production accompanying differentiation was detected visually by CM-H_2_DCFDA (Fig 1F), and the significant increase in levels were validated quantitatively using Amplex Red (Fig 1G). When F9 cells were differentiated towards a PE lineage, *Nox1* (S2A Fig) and *Nox4* (S2B Fig) transcript levels decreased significantly (*P* < 0.05) when compared to DMSO- and RA-treated F9 cells. It should be noted, however, that a significant oxidative state (*P* < 0.05; S2C Fig) was maintained, thus suggesting another potential source of ROS when cells differentiate to PE.

### *Gata6* induction results in increased NOX1 and NOX4 levels

**Fig 2:**
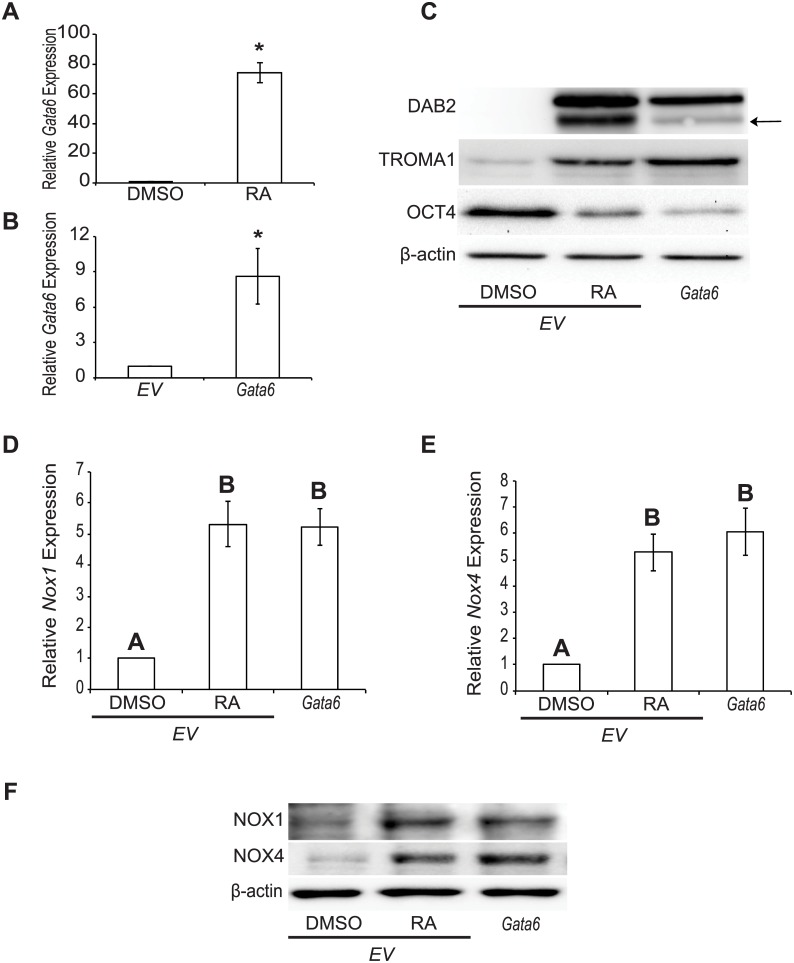
*Gata6* induction results in increased NOX1 and NOX4 levels. Total RNA and protein was collected from F9 cells treated with DMSO or RA, or transfected with pcDNA3.1-*EV* (*EV*) and treated with either DMSO or RA, or transfected with pcDNA3.1-*Gata6* (*Gata6*) and cultured 4 days. **(A)** *Gata6* expression of F9 cells treated with DMSO or RA. **(B)** *Gata6* expression of F9 cells transfected with *EV* or *Gata6*. **(C)** Immunoblot analysis for DAB2 (arrow), TROMA1 and OCT4 in F9 cells transfected with *EV* and treated with DMSO or RA or F9 cells ectopically expressing *Gata6*. **(D)** *Nox1* and **(E)** *Nox4* expression in F9 cells transfected with *EV* and treated with DMSO or RA, or F9 cells ectopically expressing *Gata6*. **(F)** Immunoblot analysis for NOX1 and NOX4 in F9 cells transfected with *EV* and treated with DMSO or RA, or F9 cells ectopically expressing *Gata6*. A total of 3 independent experiments were analyzed and results are presented as mean ± SEM. * denotes significance (*P* < 0.05) tested by a Student’s t-Test, whereas letters denote groups of significance (*P* < 0.05) tested by a One-Way ANOVA followed by a Tukey’s test.

*Gata6*, a master regulator of endoderm and extraembryonic endoderm formation, is significantly upregulated (*P* < 0.05) in F9 cells treated with RA (Fig 2A). This evidence, together with the presence of GATA-binding sites in the *Nox1* promoter [11], and that GATA6 is responsible for increased *Nox1* transcription in Caco-2 cells [12] led us to propose that GATA6 might be responsible for the upregulation of *Nox1 and Nox4* in F9 cells preceding their differentiation. To address this, we overexpressed *Gata6* and then analyzed *Nox1* and *Nox4* expression and protein levels using qRT-PCR and immunoblot analysis, respectively. When *Gata6* was overexpressed alone (Fig 2B), it induced PrE [10], as evident by the increase in the differentiation markers DAB2 and TROMA1, and the decrease in the pluripotency marker OCT4 (Fig 2C). *Gata6* overexpression also induced a significant increase (*P* < 0.05) in *Nox1* (Fig 2D) and *Nox4* (Fig 2E) expression. Interestingly, these changes were associated with an apparent increase in the levels of NOX1 and NOX4 protein (Fig 2F), which were comparable to those seen in RA-treated F9 cells. Together, results indicate that changes in NOX1 and NOX4 levels accompany F9 cell differentiation to PrE, and would suggest that the increase in ROS is due in part to these changes. Thus, studies were conducted to determine if the activity of NOX1 and/or NOX4 is/are required for PrE differentiation.

### Pan-NOX inhibition blocks RA-mediated differentiation

**Fig 3:**
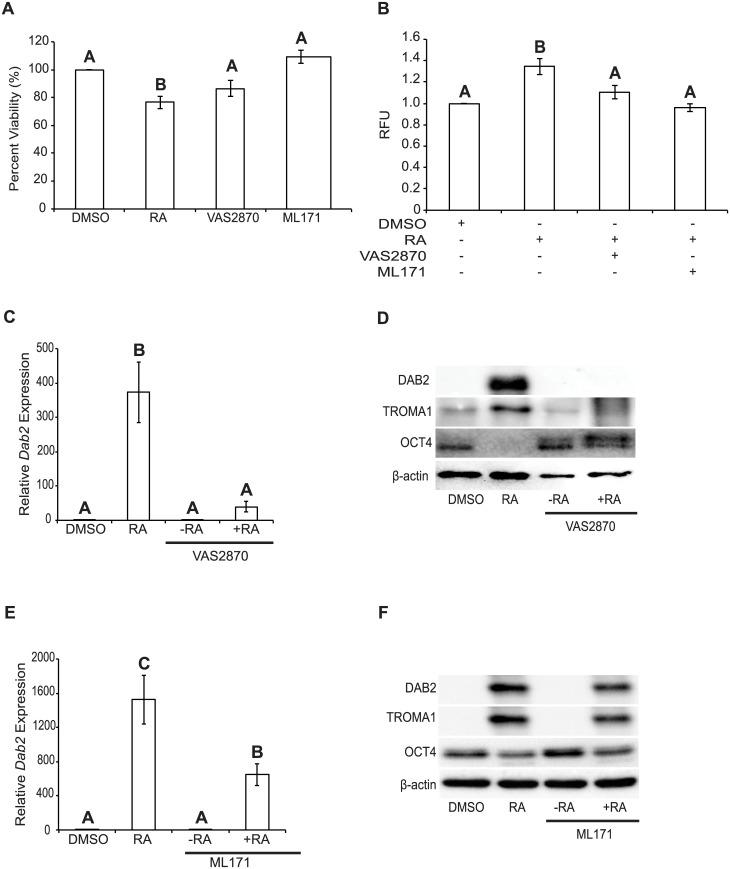
Chemical inhibition of NOX proteins attenuates RA-mediated differentiation of F9 cells. **(A)** MTT cell viability assay of F9 cells treated with DMSO, RA, VAS2870 or ML171. **(B)** F9 cells were treated with DMSO, RA, VAS2870 and RA, or ML171 and RA and assayed for ROS production using Amplex Red. **(C)** *Dab2* expression of F9 cells treated with DMSO, RA, VAS2870, or VAS2870 and RA. **(D)** Immunoblot analysis for DAB2, TROMA1, and OCT4 in F9 cells treated with DMSO, RA, VAS2870, or VAS2870 and RA. **(E)** *Dab2* expression of F9 cells treated with DMSO, RA, ML171, or ML171 and RA. **(F)** Immunoblot analysis for DAB2, TROMA,1 and OCT4 in F9 cells treated with DMSO, RA, ML171, or ML171 and RA. β-actin was used as a loading control. A total of 4 independent experiments were analyzed and results are presented as mean ± SEM. Letters denote groups of significance (*P* < 0.05) tested by a One-Way ANOVA followed by a Tukey’s test.

Our previous study revealed that Diphenyleneiodonium chloride (DPI), an inhibitor of flavoenzymes that produce ROS attenuates RA-induced PrE differentiation [3]. DPI is used frequently to inhibit ROS production, but since it acts on numerous oxidoreductases, including the NOX proteins [13] the source of the ROS may not be completely NOX-dependent. To test the hypothesis that the activity of the NOX proteins is necessary for PrE differentiation, F9 cells were treated with NOX inhibitors. First, the pan-NOX inhibitor, VAS2870 [14, 15] was used to determine specifically if members of the NOX family were necessary for F9 cells to differentiate to PrE. VAS2870 inhibits NOX1, NOX2 [15] and NOX4 [14], and although it can inhibit all NOX isoforms it does not serve as an antioxidant because it has no O_2_^-^ scavenging effect [14]. VAS2870 treatment showed no significant effect on cell viability relative to the DMSO control (Fig 3A), but it did cause a significant attenuation (*P* < 0.05) in the amount of ROS produced in cells co-treated with RA (Fig 3B). Cells treated with DMSO-, RA-, VAS2870-, or co-treated with VAS2870 and RA were examined using qRT-PCR for *Dab2* (Fig 3C), and immunoblot analysis for DAB2, TROMA1 and OCT4 (Fig 3D). Results show that *Dab2* expression, relative to the loading control *L14*, was not significantly different between DMSO and VAS2870. The expression of *Dab2* in F9 cells treated with RA alone was, as expected, significantly higher (*P* < 0.001) from the DMSO treatment, but surprisingly dramatically reduced in cells treated with RA and VAS2870 (Fig 3C). Immunoblot analysis showed comparable results with elevated DAB2 levels following RA treatment alone (Fig 3D). A similar increase was seen with the TROMA1 marker, while OCT4 levels decreased with RA treatment. The OCT4 signal, however, remained elevated in VAS2870 and RA-treated cells, and was comparable to that seen in DMSO-treated cells (Fig 3D). Thus, inhibiting NOX activity attenuates RA-mediated differentiation, which suggests strongly that NOX complexes are necessary for F9 cell differentiation.

### NOX1-specific inhibition attenuates RA-induced differentiation

F9 cells were treated with 250 nM of ML171, the IC_50_ of this NOX1-specific inhibitor [16]. ML171 has no ROS scavenging effects [16], and at this concentration it did not have an effect on F9 cell viability (Fig 3A). Co-treatment with RA, however, showed ROS levels that were significantly lower than those seen in RA-treated F9 cells (Fig 3B). qRT-PCR analysis revealed that *Dab2* expression in DMSO- and ML171-treated F9 cells were not significantly different from each other, but both were significantly different (*P* < 0.001) from that in RA-treated F9 cells, and F9 cells co-treated with RA and ML171 (*P* < 0.05) (Fig 3E). Since *Dab2* expression in cells co-treated with RA and ML171 was significantly lower (*P* < 0.05) than that in RA-treated F9 cells (Fig 3E), but not as low as that in cells co-treated with RA and VAS2870 (Fig 3C) this suggest that inhibiting NOX1 alone was not sufficient in completely attenuating *Dab2* expression. Immunoblot analysis for DAB2 and TROMA1 showed no signals in DMSO- or ML171-treated F9 cells (Fig 3F). F9 cells co-treated with ML171 and RA, however, showed DAB2 and TROMA1 signals (Fig 3F), but they appeared to be of less intensity than that seen in the RA alone lane. OCT4 signals were seen in DMSO-, ML171 and RA- and ML171-treated F9 cells, but again the intensity appeared reduced in RA-treated F9 cells with or without ML171 (Fig 3F). Since NOX4 was not targeted chemically, as there are no known specific inhibitor(s) that do not have ROS scavenging effects or additionally target other NOX proteins [16], we can only conclude that NOX proteins are required for PrE differentiation, and that NOX1 specifically plays a role in this process.

### *Nox1* and *Nox4* knockdown decreases ROS production

**Fig 4:**
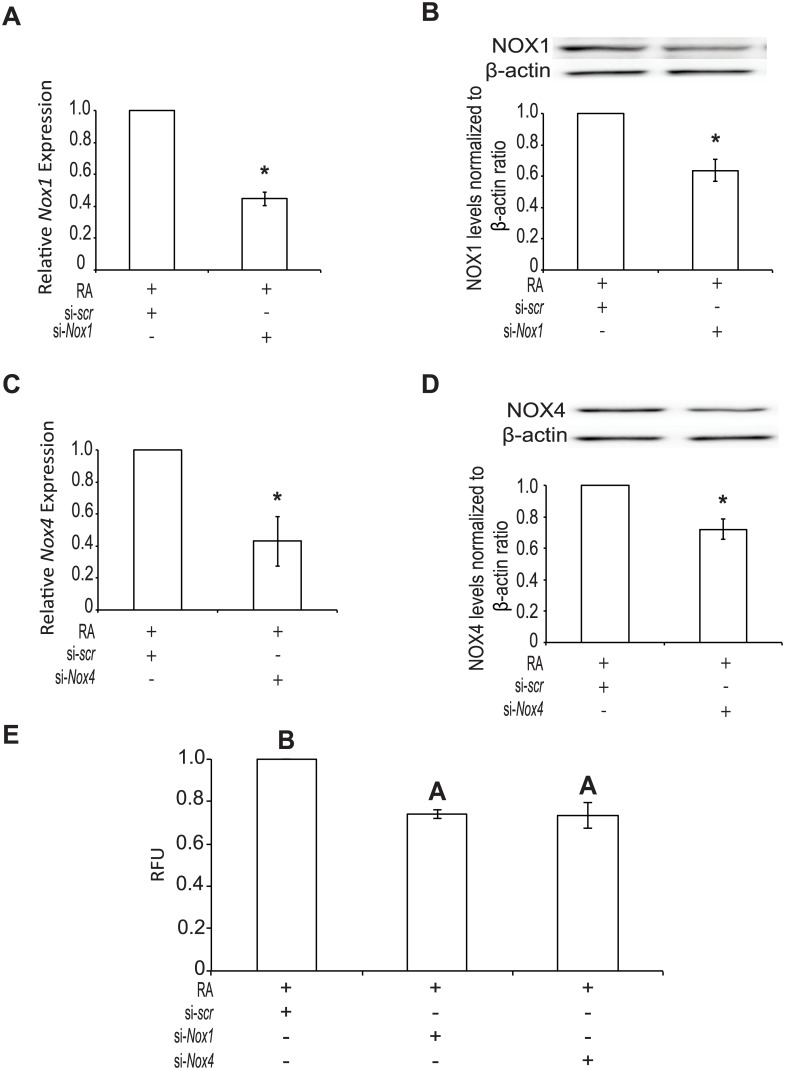
*Nox1* and *Nox4* knockdown reduces ROS production. Total RNA was collected from F9 cells transfected with scrambled (scr) si-*RNA*, si-*Nox1* and/or si-*Nox4* siRNA and cultured 4 days with RA treatment. **(A)** Expression of *Nox1* and **(B)** NOX1 protein levels following transfection with *scr* or si*-Nox1* and RA treatment. **(C)** Expression of *Nox4* and **(D)** NOX4 protein levels following transfection with *scr* or si*-Nox1* and RA treatment. **(E)** ROS production detected using Amplex Red of F9 cells transfected with *scr*, si*-Nox1*, or si*-Nox4* and treated with RA. A total of 3 independent experiments were analyzed and results are presented as mean ± SEM. * denotes significance (*P* < 0.05) tested by a Student’s t-Test, whereas letters denote groups of significance (*P* < 0.05) tested by a One-Way ANOVA followed by a Tukey’s test.

To corroborate the chemical inhibitor data, F9 cells were transfected with a siRNA vector to knockdown *Nox1*, *Nox4* expression or a scrambled siRNA and treated with DMSO or RA as negative and positive controls, respectively. Since there is a significant increase (*P* < 0.05) in *Nox1* and 4 expressions after RA treatment (Fig 1A and 1B and S1A–S1C Fig), F9 cells were treated with RA and the siRNA knockdown efficiency was compared to that seen with the RA-treated scrambled siRNA control. F9 cells containing *Nox1* (Fig 4A) and *Nox4* (Fig 4C) siRNAs showed significantly less expression of the respective *Nox* genes (*P <* 0.05) when compared to the RA-treated scrambled control. Both siRNA targeting vectors transfected into cells did not affect the individual expression of *Nox1* or *Nox4* (data not shown). The levels of NOX1 (Fig 4B) and NOX4 (Fig 4D) were examined to test if the knockdown effects were seen at the protein level. Results show that levels were significantly lower (*P* < 0.05) in cells containing the targeting siRNAs and treated with RA, compared to RA-treated F9 cells transfected with the scrambled siRNA. Furthermore, knocking down NOX1 and NOX4 in F9 cells treated with RA showed a significant reduction (*P <* 0.05) in ROS levels compared to those seen in cells transfected with the scrambled siRNA and treated with RA (Fig 4E).

### *Nox1* and *Nox4* knockdown attenuates RA-induced differentiation

**Fig 5:**
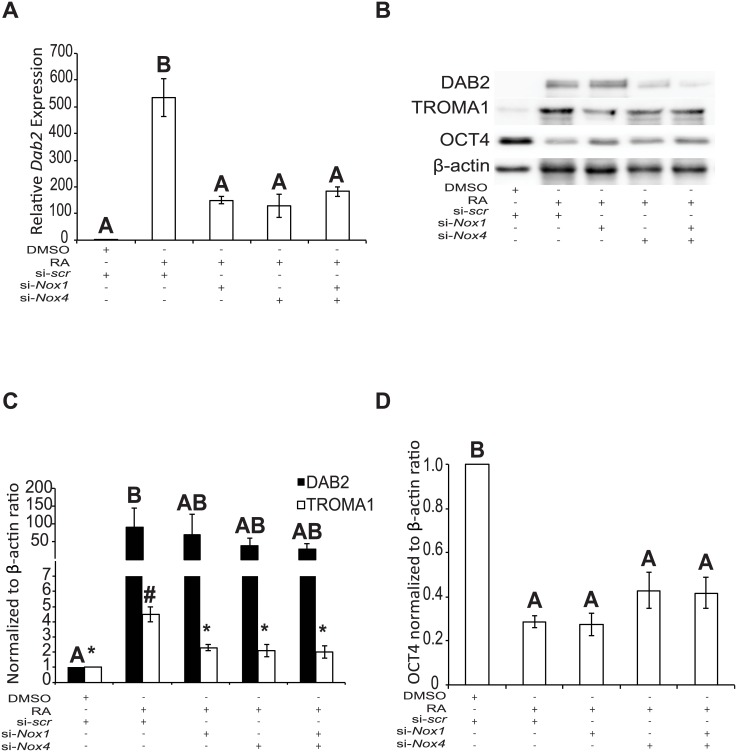
*Nox1* and *Nox4* knockdown attenuates RA-induced differentiation. RNA was collected from F9 cells transfected with scrambled (*scr*) si-*RNA*, si-*Nox1* and/or si-*Nox4* siRNA and cultured 4 days with RA treatment. **(A)** *Dab2* expression of F9 cells transfected with *scr*, si*-Nox1*, or si*-Nox4* and treated with RA. **(B)** Immunoblot analysis for DAB2, TROMA1, and OCT4 in F9 cells transfected with *scr*, si*-Nox1*, or si*-Nox4* and treated with RA. **(C)** Densitometric analysis for DAB2, TROMA1, and **(D)** OCT4 in F9 cells transfected with *scr*, si*-Nox1*, or si*-Nox4* and treated with RA. β-actin was used as a loading control. A total of 3 independent experiments were analyzed and results are presented as mean ± SEM. Letters and symbols denote groups of significance (*P* < 0.05) tested by a One-Way ANOVA followed by a Tukey’s test.

qRT-PCR and immunoblotting were used to determine if the knockdowns would affect *Dab2* expression (Fig 5A) or levels of DAB2, TROMA1, and/or OCT4 protein (Fig 5B–5D). Analysis showed that compared to RA treatment, a significant reduction (*P* < 0.05) in *Dab2* expression (Fig 5A) was seen when either *Nox1* or *Nox4* expression was knocked down. Also, there was no significant difference between the individual knockdowns compared to that due to the knockdown of both genes together. As expected, DAB2 and TROMA1 levels were low or not detected in cells transfected with the scrambled siRNA and treated with DMSO, however they did increase significantly in cells treated with RA (Fig 5B and 5C). Also, it is interesting to note that DAB2 levels in cells transfected with the targeted siRNAs and treated with RA were not significantly different between those transfected with the *scr* siRNA and treated with either DMSO or RA. However, TROMA1 levels were significantly lower in cells transfected with targeted siRNAs and treated with RA than RA treated cells transfected with *scr* siRNA (*P* < 0.05) (Fig 5C). OCT4 levels were high in the DMSO-treated *scr* siRNA control cells, but these were significantly reduced (*P* < 0.05) in F9 cells transfected with either the *scr* siRNA and treated with RA or those transfected with the siRNA targeting vectors and treated with RA (Fig 5B and 5D). Together with the inhibitor data, these results would indicate that NOX1 and NOX4 play a role in PrE differentiation.

### Overexpressing *Nox1* and *Nox4* increases ROS production

**Fig 6:**
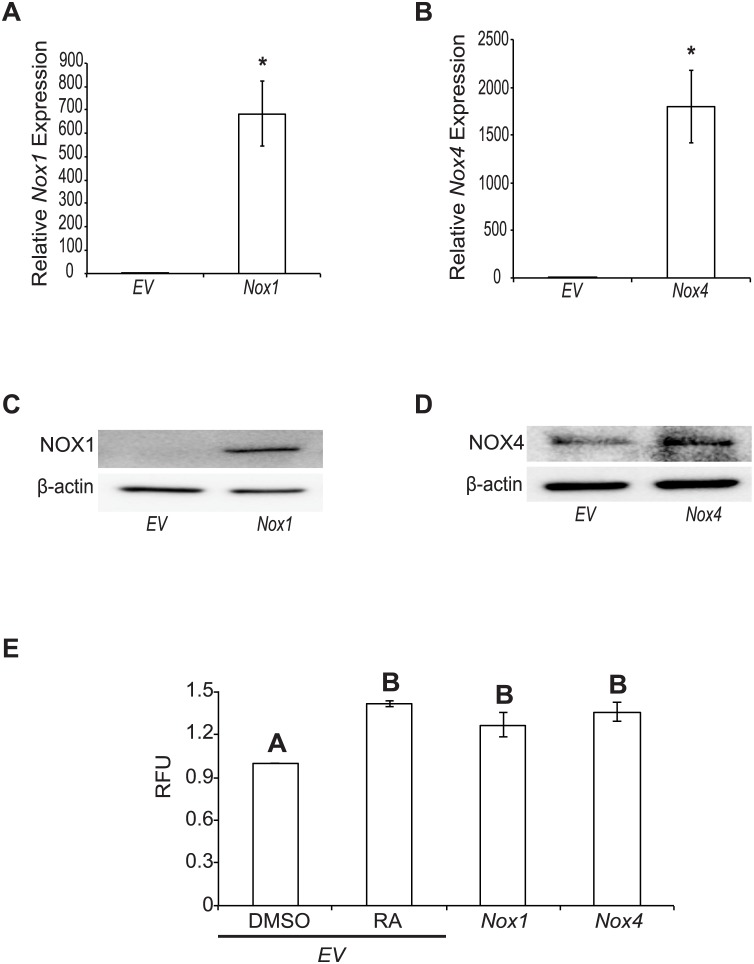
Overexpressing *Nox1* and *Nox4* increases ROS production. Total RNA was collected from F9 cells transfected with an empty vector (*EV*) control, pcDNA3.1-*Nox1* or pcDNA3.1-*Nox4* and cultured 4 days. **(A)** *Nox1* and **(B)** *Nox4* expression following transfection of pcDNA3.1-*Nox1* or pcDNA3.1-*Nox4*, respectively, and relative to *EV* transfected cells. **(C)** NOX1 and **(D)** NOX4 protein levels following transfection of pcDNA3.1-*Nox1* or pcDNA3.1-*Nox4*, respectively, relative to *EV* transfected cells. **(E)** ROS production using Amplex Red in F9 cells transfected with *EV* and treated with DMSO or RA, or transfected with pcDNA3.1-*Nox1* or pcDNA3.1-*Nox4*. A total of 3 independent experiments were analyzed and results are presented as mean ± SEM. * denotes significance (*P* < 0.05) tested by a Student’s t-Test, whereas letters denote groups of significance (*P* < 0.05) tested by a One-Way ANOVA followed by a Tukey’s test.

Since the inhibitor and genetic knockdown studies attenuated the formation of PrE, and our earlier study showing F9 cells treated with H_2_O_2_ develop into PrE [3], it seemed logical to propose that *Nox* overexpression should induce F9 cells. To test if *Nox1* or *Nox4* overexpression induced differentiation, F9 cells were transfected with pcDNA3.1-*EV* and treated with either DMSO or RA or transfected with pcDNA3.1-*Nox1* or *-Nox4*. The relative degree of overexpression was determined by qRT-PCR, and results showed an approximate 700 and 1800-fold increase for *Nox1* (Fig 6A) and *Nox4* (Fig 6B), respectively. Similarly, NOX1 and NOX4 protein levels were higher in F9 cells transfected with the respective *Nox* construct (Fig 6C and 6D). Having established that transfection resulted in elevated NOX1 and NOX4 levels, we examined if this would affect ROS levels. For the positive control, F9 cells transfected with pcDNA3.1-*EV* and treated with RA showed a significant increase (*P* < 0.05) in ROS levels over those seen in transfected cells treated with DMSO (Fig 6E). Note that the levels seen following RA treatment were comparable to those in cells transfected with either *Nox1* or *Nox4*.

### *Nox* overexpression activates canonical Wnt signaling, but does not promote differentiation

**Fig 7:**
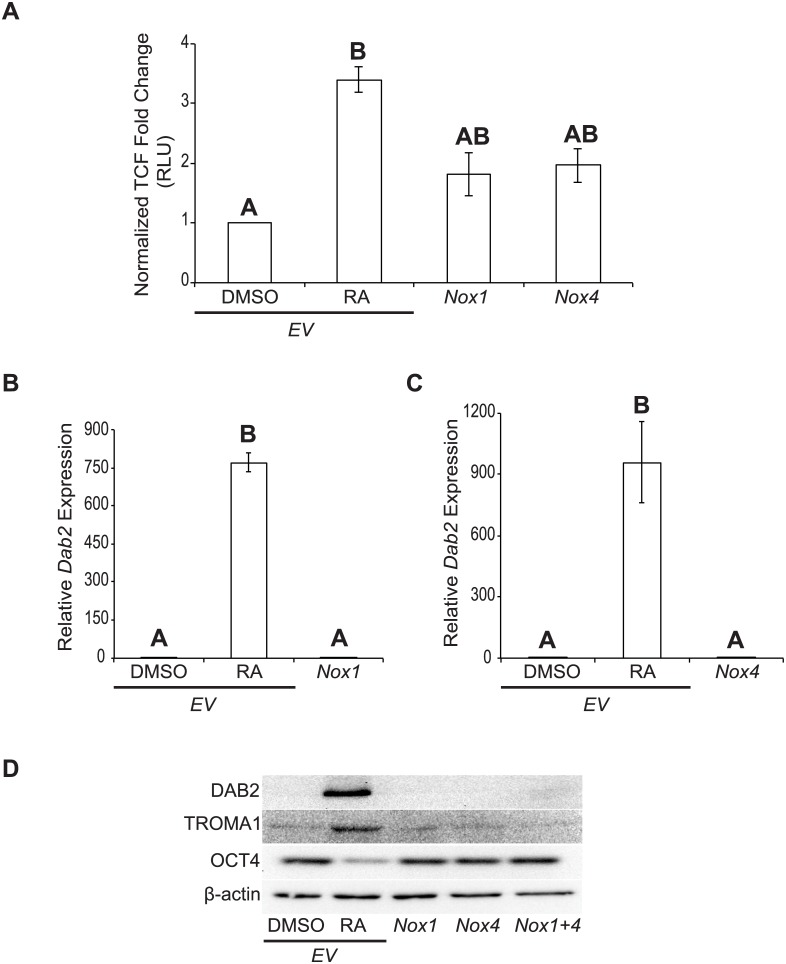
*Nox* overexpression activates canonical Wnt signaling, but does not promote differentiation. **(A)** Dual-luciferase assay of TCF activity in F9 cells transfected with *EV* and treated with DMSO or RA, or transfected with pcDNA3.1-*Nox1* or pcDNA3.1-*Nox4*. **(B)** *Dab2* expression in F9 cells transfected with *EV* and treated with DMSO or RA, or transfected with pcDNA3.1-*Nox1*. **(C)** *Dab2* expression in F9 cells transfected with *EV* and treated with DMSO or RA, or transfected with pcDNA3.1-*Nox4*. **(D)** Immunoblot analysis for DAB2, TROMA1, and OCT4 in F9 cells transfected with *EV* and treated with DMSO or RA, or transfected with pcDNA3.1-*Nox1*, pcDNA3.1-*Nox4* or both vectors. β-actin was used as a loading control. A total of 3 independent experiments were analyzed and results are presented as mean ± SEM. Letters denote groups of significance (*P* < 0.05) tested by a One-Way ANOVA followed by a Tukey’s test.

The overexpression results and those from earlier studies [3, 4] suggested that the increased levels in ROS due to ectopic *Nox1* or *Nox4* expression would activate canonical Wnt-β-catenin signaling. To test this F9 cells were co-transfected with *Nox1* or *Nox4*, and the firefly luciferase pGL3-*BARL* reporter that is used as readout for active Wnt-β-catenin signaling [17]. All cells were transfected with the *Renilla* luciferase construct, TK-*RL* to normalize for luciferase expression. RA-treated F9 cells transfected with pcDNA3.1-*EV* showed a significant increase (*P* < 0.05) in TCF activity compared to the DMSO control (Fig 7A). F9 cells overexpressing *Nox1* or *Nox4* also showed increased TCF activity, however, this increase was less than that seen in RA-treated F9 cells, and was not significant from either RA or DMSO treatments (Fig 7A). Nevertheless, F9 cells overexpressing *Nox1* or *Nox4* were analyzed for PrE differentiation, and as expected *Dab2* expression relative to the *L14* loading control was significantly higher (*P* < 0.001) in RA-treated F9 cells than that in DMSO treated cells (Fig 7B and 7C). *Dab2* expression resulting from either the overexpression of *Nox1* (Fig 7B) or *Nox4* (Fig 7C) was not significantly different from that in DMSO-treated F9 cells, suggesting that differentiation had not occurred. Similarly, DAB2 protein was only present in RA-treated cells, and this evidence of differentiation was seen in the corresponding increase in TROMA1 and concomitant decrease in OCT4 levels (Fig 7D). TROMA1 signals were not seen in DMSO-treated, *Nox1*-, *Nox4*-, or *Nox1* and *Nox4*-overexpressing cells, and the appearance of OCT4 in the same samples was indicative that PrE had not differentiated as a result of *Nox1* and/or *Nox4* overexpression (Fig 7D). Thus, even though overexpressing *Nox1* or *Nox4* in F9 cells increased ROS levels, there was no significant activation of Wnt-β-catenin signaling or hallmark molecular indicators of PrE to suggest that the overexpression of *Nox1*, *Nox4* or both was sufficient to induce differentiation.

## Discussion

ROS, which are by-products of metabolic processes produced through the incomplete reduction of oxygen, create oxidative stress on cells, and damage nucleic acids, proteins and lipids. Although this damage is linked to age-related and other human disease pathogenesis, recent studies now show cells benefitting from ROS as a second messenger to modulate biological processes such as differentiation, apoptosis and proliferation [18, 19]. F9 cells are induced to form primitive endoderm when treated with RA, an *in vitro* process that mimics a very early event in mouse embryogenesis. Differentiation is accompanied by numerous changes in gene expression and morphology and requiring several signaling pathways. One of these pathways involves canonical Wnt-β-catenin signaling [1], where *Wnt6* is induced by GATA6 [10] a master regulator of extraembryonic and embryonic endoderm. Regulation of this Wnt pathway occurs in a redox-regulated manner [3] involving NRX and DVL, the latter serving as a lynchpin in the three major Wnt signaling pathways [4]. We have shown previously that cytosolic ROS plays a role in F9 cell differentiation to form primitive endoderm [3]. ROS impact positively on the canonical Wnt-β-catenin signaling pathway, and the *Nox* genes that are upregulated in response to RA were identified as candidates responsible for this cytosolic ROS [3]. Increases in mitochondrial ROS have been also observed in F9 cells differentiated to primitive and parietal endoderm, a consequence of what appears to be a metabolic transition from aerobic glycolysis towards oxidative phosphorylation (manuscript in preparation). This increase in mitochondrial ROS might explain the maintained oxidative state of F9 cells at PE, despite the reduction in *Nox1* and *Nox4* expression (S2A and S2B Fig).

The expression profiles of the six mouse *Nox* genes led us to interrogate *Nox1* and *Nox4*, which were the most up-regulated in response to RA in F9 cells [3]. In addition to RA (Fig 1A–1D), both genes are up-regulated, and the level of the corresponding proteins increase in response to GATA6 (Fig 2D–2F). Previous studies provide evidence for a link between GATA6 and *Nox* genes, specifically *Nox1*, which is activated directly by GATA6 [11, 12]. NOX1 impacting on Wnt signaling occurs in colonic and intestinal epithelial cells [8, 20], and it is known to post-transcriptionally increase the levels of Keratin 18 [21], a cytokeratin encoded by transcripts that increase during RA-induced F9 cell differentiation [22]. Furthermore, NOX1 and NOX4 activate the p38 MAPK pathway in mouse and human cells [23, 24], which is also required to inhibit GSK-3β thereby maintaining Wnt in the on-state during PrE differentiation [25]. Evidence for NOX4 interacting with Wnt signaling or regulated by GATA transcription factors is not as compelling as NOX1. However, it is interesting to note a study showing the opposite scenario exists in cardiomyocytes, where NOX4 increases *Gata4* expression [26]. Other reports have linked NOX4 to cell proliferation, cytoskeletal reorganization, migration and differentiation [27–30], which incidentally are processes that F9 cells participate in during differentiation [31].

Having identified that NOX1 and NOX4 were under the indirect or direct control of GATA6, we next used chemical inhibitors to test whether or not the activity of both NOX proteins were required for PrE differentiation. As noted above, a previous study in our lab using the non-specific flavoenzyme inhibitor DPI, demonstrated the necessity for ROS in the differentiation of F9 cells [3]. Since DPI acts on other non-NOX flavoenzymes [13], we refined the approach and used a pan-NOX and NOX1-specific inhibitor to test their effects on differentiation (Fig 3). Both inhibitors affected differentiation at the gene and protein levels, but VAS2870, the pan-NOX inhibitor was more potent than ML171, the selective inhibitor of NOX1 activity (Fig 3). Although there could be many reasons to explain this, the fact that all the NOX proteins were being inhibited by VAS2870 would suggest that multiple NOX proteins contribute to the ROS required for differentiation. This argument would be tested later as these pharmacological inhibitor studies proved informative, but required a genetic approach to corroborate the data. Mouse knockout models exist for all 6 *Nox* genes [14, 32, 33], but disrupting any one of them individually or in combination does not appear to have obvious effects in embryos. Therefore, an alternative strategy was to use siRNAs, which have had some success in knocking down the expression of specific *Nox* genes [14]. This approach was used to knockdown *Nox1* and/or *Nox4* gene expression, and it resulted in reduced expression and protein levels of NOX1 and NOX4 (Fig 4A–4D). Knockdown led to the attenuation of differentiation, as evident by the decrease in the markers of PrE, and the maintenance of pluripotency as noted by the persistence of the OCT4 signal (Fig 5A–5D). Together with the inhibitor results, this data adds support that NOX activity, specifically from NOX1 and NOX4, is necessary for F9 cells to form PrE. While encouraged by these results, the contribution to the differentiation process by the two other *Nox* genes and *Duox2* that are upregulated by RA in F9 cells needs to be considered and is currently under investigation.

Although NOX1 and NOX4 are members of the NOX subfamily that require p22^phox^, the former generates primarily superoxide, while the major product of the latter is H_2_O_2_ [34]. *Nox4* is widely expressed in human, while *Nox1* is much more restricted [32]. Nevertheless, there is overlap in places where functional redundancy might explain why the *in vivo* knockout models do not show obvious phenotypes. This does not appear to be the case for the F9 *in vitro* cell model as there was a common effect seen when knocking down either *Nox1* or *Nox4*. Thus, it is possible that attenuating the activity of one NOX isoform *in vitro* reduces the total amount of ROS required for differentiation, and this cannot be compensated by the other members. The overexpression of either *Nox1* and/or *Nox4* (Fig 6A–6D) in F9 cells nevertheless resulted in increased ROS levels (Fig 6E), and what appeared to be an increase in canonical Wnt signaling (Fig 7A). However, these increases were not sufficient to induce differentiation as seen following the application of exogenous H_2_O_2_ [3]. Although there is much debate centered on the recently reported *Nox1*, *Nox2* and *Nox4* triple knockout mouse model [33], our information would indicate that ROS produced individually by at least two of the NOX isoforms is necessary in the F9 cell model of primitive extraembryonic endoderm formation. Building from previous studies our model now adds to the signaling hierarchy and crosstalk within these networks where RA induces *Gata6* expression, which in turn regulates several genes including *Wnt6* [10], *Nox1* and *Nox4* (this study). The subsequent increase in ROS permits NRX to dissociate from DVL, thereby priming the Wnt pathway [4] prior to the appearance of the WNT6 ligand that is sufficient to promote PrE differentiation [1]. WNT6 signaling is attenuated in a negative feedback loop involving Dickkopf-1 [35], and this is required for PrE cells to complete their differentiation to PE [36]. Given the report showing canonical Wnt signaling activates RAC1 and this in turn increases RAC1-dependent NOX1 activity that produces the ROS needed to oxidize NRX and dissociate it from DVL in order to keep the pathway in the on-state [8], it is tempting to speculate that a similar mechanism occurs in F9 cells in response to RA. However, since NOX4 is not RAC1-dependent [9], and is required for PrE differentiation (this study), canonical Wnt signaling must be stimulating NOX4 activity in another manner. Given that the production of ROS is increased as a direct result of increased NOX4 expression [37], the possibility exists that increased NOX4 activity is the result of an upregulation in the *Nox4* gene by active Wnt signaling. Towards that end, experiments are underway to test if the *Nox* genes are WNT target genes or simply downstream in the pathway. Whatever the case, that aberrant Wnt signaling is prevalent in cancer biology [38, 39] underpins the importance of better understanding the crosstalk imparted by NOX proteins and ROS on the Wnt-β-catenin signaling pathway in both development and cancer research.

## Supporting information

S1 Fig *Nox1* and *Nox4* are upregulated during RA-treatment. Total RNA was collected from F9 cells treated with DMSO or RA for 4 days. **(A)** Ct values for *Nox1* and *Nox4* under DMSO or RA treatment. **(B and C)** Graphical illustration of Ct values of *Nox1* and *Nox4* under DMSO or RA treatment over 40 cycles. *L14* served as a control.
(EPS)

S2 Fig F9 cells show reduction in *Nox1* and *Nox4* expression yet maintain high oxidative state at PE. Total RNA was collected from F9 cells induced to differentiate into PrE and PE. Expression of *Nox1* **(A)** and *Nox4* **(B)** following DMSO, RA, and RA with db-cAMP treatment. **(C)** Oxidative state of F9 cells following treatment with DMSO, RA, RA with db-cAMP. A total of 4 independent experiments were analyzed and results are presented as mean ± SEM. Letters denote groups of significance (*P* < 0.05) tested by a One-Way ANOVA followed by a Tukey’s test.
(EPS)
